# Identification of Individual Target Molecules Using Antibody-Decorated DeepTip^TM^ Atomic-Force Microscopy Probes

**DOI:** 10.3390/biomimetics9040192

**Published:** 2024-03-22

**Authors:** Daniel Corregidor-Ortiz, Rafael Daza, Luis Colchero, Raquel Tabraue-Rubio, José Miguel Atienza, Manuel Elices, Gustavo V. Guinea, José Pérez-Rigueiro

**Affiliations:** 1 Departamento de Ciencia de Materiales, ETSI Caminos, Canales y Puertos, Universidad Politécnica de Madrid, 28040 Madrid, Spain; daniel.corregidor@ctb.upm.es (D.C.-O.); rafael.daza@upm.es (R.D.); raquel.tabraue@ctb.upm.es (R.T.-R.); josemiguel.atienza@upm.es (J.M.A.); m.elices@upm.es (M.E.); gustavovictor.guinea@ctb.upm.es (G.V.G.); 2 Center for Biomedical Technology (CTB), Universidad Politécnica de Madrid, Pozuelo de Alarcón, 28223 Madrid, Spain; 3 Bioactive Surfaces S.L., C/Puerto de Navacerrada 18, 28260 Galapagar, Spain; luis.colchero@ctb.upm.es; 4 Biomedical Research Networking Center in Bioengineering, Biomaterials and Nanomedicine (CIBER-BBN), 28029 Madrid, Spain; 5 Biomaterials and Regenerative Medicine Group, Instituto de Investigación Sanitaria del Hospital Clínico San Carlos (IdISSC), C/Prof. Martín Lagos s/n, 28040 Madrid, Spain

**Keywords:** affinity atomic-force microscopy, functionalization, single-molecule resolution, antibody, antigen

## Abstract

A versatile and robust procedure is developed that allows the identification of individual target molecules using antibodies bound to a DeepTip^TM^ functionalized atomic-force microscopy probe. The model system used for the validation of this process consists of a biotinylated anti-lactate dehydrogenase antibody immobilized on a streptavidin-decorated AFM probe. Lactate dehydrogenase (LDH) is employed as target molecule and covalently immobilized on functionalized MicroDeck^TM^ substrates. The interaction between sensor and target molecules is explored by recording force–displacement (F–z) curves with an atomic-force microscope. F–z curves that correspond to the genuine sensor–target molecule interaction are identified based on the following three criteria: (i) number of peaks, (ii) value of the adhesion force, and (iii) presence or absence of the elastomeric trait. The application of these criteria leads to establishing seven groups, ranging from no interaction to multiple sensor–target molecule interactions, for which force–displacement curves are classified. The possibility of recording consistently single-molecule interaction events between an antibody and its specific antigen, in combination with the high proportion of successful interaction events obtained, increases remarkably the possibilities offered by affinity atomic-force microscopy for the characterization of biological and biomimetic systems from the molecular to the tissue scales.

## 1. Introduction

Since atomic-force microscopy was developed [1], it has been extensively used for topographic and mechanical analyses of samples of the most varied nature. In particular, its application to biological and biomimetic systems takes advantage of its unique combination of a spatial resolution in the range of nanometers and a force resolution in the range of piconewtons. Additionally, biological and biomimetic samples may be observed under physiological conditions. As a consequence of this combination of singular features, the number of AFM studies on these systems has increased steadily. Thus, AFM has allowed, for instance, the measurement of the elastic and viscoelastic properties of various biological systems [2,3,4].

The combination of this approach with the decoration of AFM probes through the binding of specific molecules has led to a new dimension in the applications available within the biological and biomimetic fields. This way, it has been possible to measure interactions between cells and viruses [5,6,7] or between pairs of molecules [8,9], or even to determine the forces that appear during the unfolding of a single macromolecule [10,11].

These experiments, however, sometimes overlook the importance of using functionalized AFM probes to which sensor molecules may be reliably bound through an efficient crosslinking chemistry. Thus, the functionalization of the probes tends to rely on a series of protocols that often lead to a relatively low reproducibility [12,13,14,15]. In particular, this low reproducibility contributes to the small proportion of successful events in which a genuine interaction between the target molecule and the sensor molecule is observed, which is typically estimated to be between 1% and 0.1% of the total number of recorded F–z curves [16,17]. In this context, the amine-functionalized DeepTip^TM^ AFM probes have shown their adequacy for the measurement of single-molecule recognition events in systems ranging from high [18] to low adhesion forces [19], while yielding percentages of successful recognition events in excess of 30%.

In this work, a robust and versatile procedure is developed that allows the decoration of DeepTip^TM^ probes with biotinylated antibodies, opening an extremely broad field in terms of the target molecules that can be identified. Antibodies are conventionally used as sensor molecules in various analytical techniques, such as the ELISA [20], Western blot [21], and affinity chromatography [22] techniques, for the specific detection of molecules of interest. Additionally, antibodies are often used in biosensors, such as in lateral flow immunoassays for the detection of COVID-19 or flu infections [23]. The interest of employing antibodies for the specific detection of target molecules with an atomic-force microscope has been highlighted in some studies, including, for instance, the detection of ferritin immobilized on gold substrates [24], the analysis of the formation of immune complexes adsorbed on mica [25], or the interaction between human serum albumin and an anti-HAS antibody [26]. In this work, a protocol is presented that allows the binding of biotinylated antibodies to DeepTip^TM^ probes. The efficiency of this process is validated with a model system in which anti-LDH decorated probes are used to identify the presence of LDH on a substrate through the recording of F–z curves.

## 2. Materials and Methods

### 2.1. Materials

The following materials were used in this study: sulfo-LC-SPDP (sulfosuccinimidyl 6-(3′-(2-pyridyldithio)propionamido)hexanoate; ThermoScientific, Waltham, MA, USA, 21650), TCEP-HC (Tris(2-carboxyethyl)phosphine hydrochloride Thermo Scientific, Waltham, MA, USA, 20490), EDTA (ethylenediaminetetraacetic acid; Sigma Aldrich, Burlington, MA, USA, E9884), MicroDeck^TM^-G-150 surfaces (Bioactive Surfaces, Galapagar, Madrid, Spain), lactate dehydrogenase (LDH; Sigma Aldrich, Burlington, MA, USA, L1006-12.5KU), biotinylated anti-lactate dehydrogenase (anti-LDH) antibodies (IgG polyclonal antibody; Origene, Rockville, MD, USA, AP21329BT-N), poly(ethylene glycol)-(N-hydroxysuccinimide 5-pentanoate) ether 2-(biotinylamino)ethane (NHS–PEG–biotin; Sigma Aldrich, Burlington, MA, USA, 757799-100μγ), streptavidin (Sigma Aldrich, Burlington, MA, USA, S4762-5 mg), DeepTip^TM^ SiN R11 atomic-force microscopy probes (Bioactive Surfaces, Galapagar, Madrid, Spain), Goat Anti-Rabbit IgG H&L Alexa Fluor^®^ 488 (Abcam, Cambridge, UK, ab150077), 5-iodoacetamidofluorescein (5-IAF; Thermo Scientific, Waltham, MA, USA, 62246), sodium pyruvate (P2256 Sigma-Aldrich, Burlington, MA, USA,), β-nicotinamide adenine dinucleotide (NADH; N8129-500 mg), and glutaraldehyde (50 wt. % in H_2_O, Sigma Aldrich, Burlington, MA, USA, 340855).

### 2.2. Functionalization of MicroDeck^TM^ Substrates with Sulfo-LC-SPDP

MicroDeck^TM^-G-150 substrates functionalized with amine groups were supplied by Bioactive Surfaces. In order to bind LDH to the substrates covalently, the crosslinking agent sulfo-LC-SPDP was used. This compound reacts with the amino groups on the surface of the substrates through the NHS (N-hydroxysuccinimide) group, leaving free pyridyl thiol groups capable of reacting with the free thiols of the cysteines. For this purpose, the following protocol adapted from [27] was used: First, the MicroDeck^TM^ substrates were incubated for 30 min in 0.1 M carbonate–bicarbonate buffer (12.5 mM NaHCO_3_, 87.5 mM Na_2_CO_3_; pH 9.1) at room temperature in order to deprotonate the amino groups. The carbonate–bicarbonate buffer was then removed, and the surfaces were incubated in a 2.5 mg/mL solution of sulfo-LC-SPDP in PBS-EDTA (8 mM Na_2_HPO_4_ and 2 mM KH_2_PO_4_, 137 mM NaCl and 2.7 mM KCl, 1 mM EDTA; pH 7.4) for 1 h at room temperature. At the end of the incubation time, the surfaces were washed extensively with PBS-EDTA.

The functionality of the piridyl groups was assessed through fluorescence microscopy using 5IAF as the specific fluorophore. MicroDeck^TM^ substrates decorated with sulfo-LC-SPDP were incubated in a 3 mg/mL solution of TCEP in PBS-EDTA at pH 7.4 for 15 min at room temperature in order to release the pyridyl thiol group. Subsequently, the substrates were washed thoroughly with PBS-EDTA at a pH of 7.4 and incubated in a solution of 1 mg/mL 5IAF (a fluorophore specific for the thiol groups) in PBS-EDTA at pH 7.4 for 1 h at RT. Finally, the surfaces were washed with 10% SDS for 5 min, then washed three times with PBS-EDTA for 5 min each time, and finally rinsed with mQ water. Samples were observed using an inverted fluorescence microscope (Leica DFC340FX) with the following acquisition parameters: exposition time 598 ms, gain 2, gamma 0.60, and magnification ×20. Functionalized (but not decorated) MicroDeck^TM^ surfaces, either as received or after being immersed in 1 mL of a glutaraldehyde solution, were used as controls.

### 2.3. Decoration of Sulfo-LC-SPDP Functionalized Substrates with LDH

Sulfo-LC-SPDP functionalized substrates were incubated in an LDH solution in PBS at two different concentrations: 400 μg/mL and 125 μg/mL overnight at 4 °C. At the end of the incubation time, the surfaces were washed with PBS-Tween 20 0.05% under gentle agitation 5 times, for 1 min each time, changing the washing solution each time. The prepared surfaces were used either for enzymatic assays or for affinity microscopy assays. In particular, to assess the activity of the LDH bound to the substrate, an enzymatic assay was performed. LDH-decorated substrates were immersed in 1 mL of a solution containing 180 μM NADH and 2.9 mM sodium pyruvate in PBS at pH 7.4. The absorbance of the supernatant at 340 nm was measured at the beginning of the assay and after an incubation of 8 h at RT, using a variation of the procedure used for assessing the presence of LDH on solid substrates [28] through the decrease in the concentration of NADH in the solution. Apart from assessing the functionality of LDH, no specific attempt was undertaken to identify the quaternary structure of the immobilized protein as either a monomer or in its common homotetrameric structure.

### 2.4. Functionalization of DeepTip^TM^ AFM Probes with Anti-LDH Antibodies

The DeepTip SiN R11 probes were decorated with biotinylated polyclonal anti-LDH antibodies. Firstly, the chips were incubated in 0.1 M of carbonate buffer at pH 9.1 for 30 min at RT. Then, they were incubated in a 2.7 mg/mL solution of NHS–PEG–biotin in PBS with a pH of 7.4 for 30 min at RT. The chips were then washed extensively with the PBS at pH 7.4 and incubated again in a 500 μg/mL solution of streptavidin in PBS at pH 7.4. The samples were then washed with PBS at pH 7.4 extensively to remove any unbound streptavidin and subsequently incubated with a biotinylated polyclonal anti-LDH antibody solution at a concentration of 0.25 mg/mL in PBS at pH 7.4 for 1 h at room temperature. Finally, the samples were washed extensively to remove any unbound antibody.

To determine the presence of anti-LDH antibodies bound to the AFM probes, an anti-anti-LDH secondary antibody (Goat Anti-Rabbit Alexa Fluor 488) was used. The chips decorated with the anti-LDH antibody were incubated in a 1:800 secondary antibody solution in 0.1% BSA-PBS at pH 7.4 for 1 h at room temperature. Samples were then washed abundantly with PBS at pH 7.4. An inverted fluorescence microscope (Leica DFC340FX) was used for image acquisition with the following observation parameters: exposition time 1 s, gain 2.1, gamma 0.83, and magnification X20. Probes not decorated with the anti-LDH antibody were employed as controls.

### 2.5. Affinity Microscopy Assays

To measure the presence of LDH in the sample, a Nanolife atomic-force microscope (Nanotec Electronica S.L., Madrid, Spain) operated in the lithography mode was used and DeepTip^TM^ SiN R11 tips (k = 0.01 N/m; resonance frequency = 11 kHz) decorated with anti-LDH antibodies were prepared as mentioned above. Measurements were carried out in PBS with a pH of 7.4 and a total of 1040 F–z curves were acquired across three independent experiments using the following parameters: contact force: 600–800 pN, contact time 1 s, approach speed 1000 nm/s, retraction speed 500 nm/s. The curves obtained were analyzed using WSxM 5.0 software [29] and a Matlab routine developed in the group.

## 3. Results and Discussions

### 3.1. Fluorescence Assays

#### 3.1.1. MicroDeck^TM^ Decorated with Sulfo-LC-SPDP

**Figure 1:**
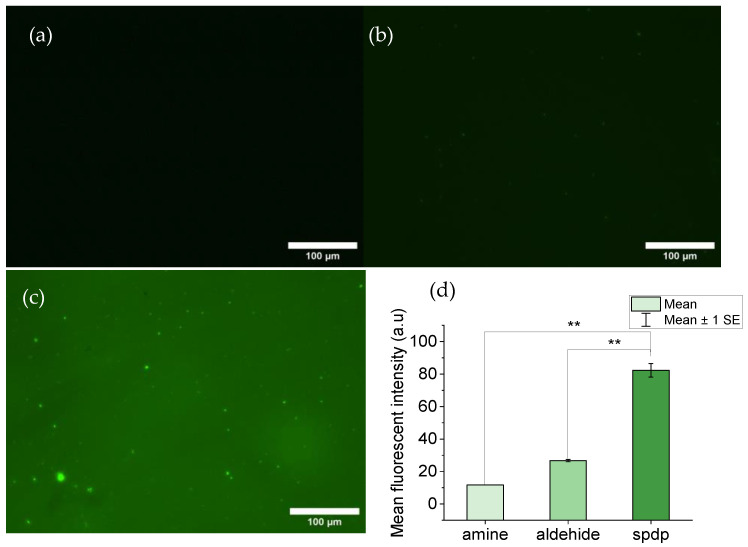
Florescence images of MicroDeck^TM^ surfaces: (**a**) Control substrate functionalized with amine groups. (**b**) Control substrate decorated with aldehyde groups. (**c**) Substrate decorated with thiol groups after incubation of a sulfo-LC-SPDP-decorated sample with TCEP. (**d**) Bar chart comparing the mean fluorescence intensities of the decorated and control samples. Statistical significance: *p* < 0.01 (**).

The efficiency of the decoration of MicroDeck^TM^ substrates with sulfo-LC-SPDP was assessed through fluorescence microscopy. Substrates decorated with sulfo-LC-SPDP were incubated with TCEP to release the piridyl group, leaving a reactive thiol group. The reactive thiol group, in turn, may bind the thiol-specific fluorophore 5IAF. MicroDeck^TM^ substrates not decorated with sulfo-LC-SPDP and MicroDeck^TM^ substrates decorated with glutaraldehyde were used as controls (Figure 1a and Figure 1b, respectively). Fluorescence intensity was measured with the ImageJ program and a statistical test was performed to corroborate whether the difference between the sulfo-LC-SPDP-decorated and control substrates was significant. As displayed in Figure 1, a clear difference between the sulfo-LC-SPDP-decorated (Figure 1c) substrate and both controls was observed. A quantification of the fluorescence intensity from these micrographs with ImageJ supports a significant statistical difference (*p* < 0.01) between them (Figure 1d).

#### 3.1.2. DeepTip^TM^ AFM Probes Decorated with anti-LDH Antibodies

**Figure 2:**
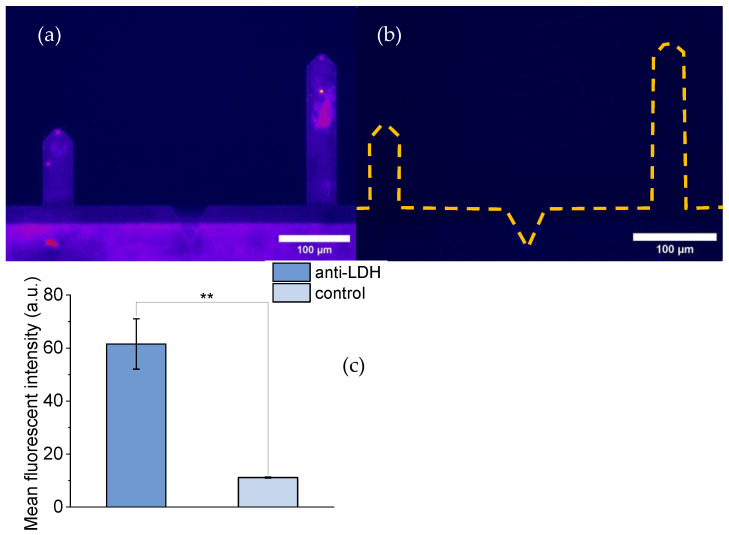
Florescence images of DeepTip^TM^ probes: (**a**) Probes decorated with an anti-LDH antibody and incubated with a fluorescent anti-anti-LDH antibody. (**b**) Control probes not decorated with an anti-LDH antibody, but incubated with a fluorescent anti-anti-LDH antibody. The broken lines correspond to the silhouette of the cantilevers (**c**) Bar chart comparing the mean fluorescent intensity of the decorated and non-decorated samples. Statistical significance: *p* < 0.01 (**).

Figure 2 compares the fluorescence images of a DeepTip^TM^ AFM probe decorated with anti-LDH antibodies (Figure 2a) and a control probe (Figure 2b). The control (not decorated with anti-LDH antibodies) probe was subjected to the same protocol as the decorated DeepTip^TM^ probe, including incubation with the secondary anti-anti-LDH antibody.

The difference in fluorescence between both samples is apparent from Figure 2. This difference was further quantified by measuring the fluorescence intensity with the ImageJ program and the results showed a statistical difference of *p* < 0.01 as estimated with the t-Student test.

### 3.2. Enzymatic Assay

**Figure 3:**
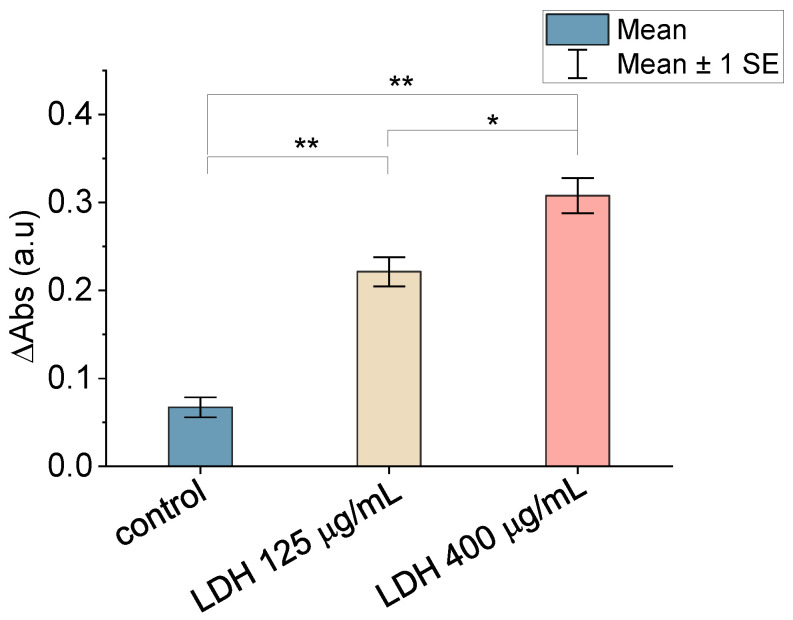
Comparison of the mean 340 nm absorbance variation of non-decorated (physical adsorption) and LDH-decorated surfaces. Statistical significance: *p* < 0.05 (*), *p* < 0.01 (**).

The results of the enzymatic assay used to assess the activity of the LDH immobilized on the MicroDeck^TM^ substrates are summarized in Figure 3. As can be seen in this figure, there is significant variation in the 340 nm absorption between the control sample (in which LDH may appear as a result of its physical adsorption to the surface) and the samples in which the enzyme was immobilized using the sulfo-LC-SPDP crosslinker. The sample incubated with a higher concentration of LDH (400 μg/mL) shows an increase in absorbance compared with the lowest concentration (125 μg/mL), and both samples, in turn, show a significantly higher absorbance than the control sample. This result confirms the covalent binding of the enzyme to the substrate and, in addition, shows that covalent immobilization to the substrate does not lead to the loss of its enzymatic activity. No attempt was pursued to obtain a monolayer of LDH on the surface or to produce a proper calibration curve in terms of the immobilized LDH as a function of the LDH concentration in the solution. In this regard, a comparison of this study’s measured values at 340 nm absorption with those presented in a previous work [28] on the detection of LDH on solid surfaces through enzymatic tests indicated that a significant fraction of the surface was decorated with LDH proteins with both concentrations. It is worth highlighting that the utilization of the enzymatic test is more discriminating for the assessment of the presence of functional LDH on the substrate than an atomic-force microscopy topographic micrograph, such as the one shown in Supplementary Figure S1.

### 3.3. Affinity Microscopy Assay

**Figure 4:**
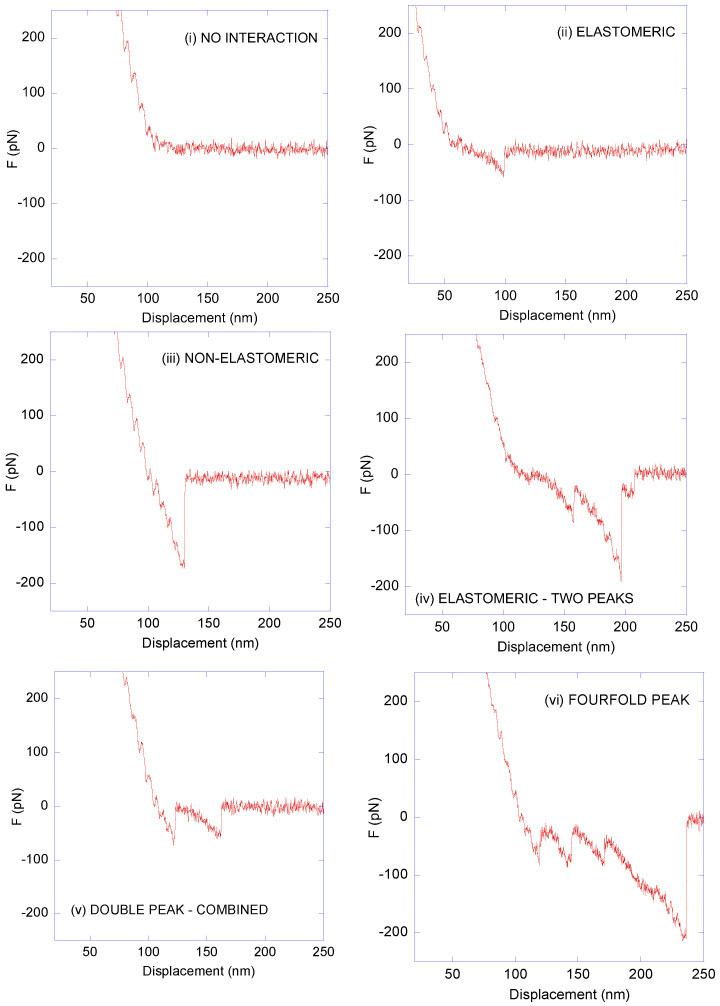
Representative force–displacement (F–z) curves obtained from the interaction between anti-LDH antibodies and LDH. In this figure, six types are shown: (**i**) no−interaction curve; (**ii**) elastomeric curve; (**iii**) non−elastomeric curve; (**iv**) elastomeric curve—two peaks; (**v**) double-peak curve—combined; (**vi**) three- or four-peak curve. An additional 7% of the curves were discarded due to them exhibiting an abnormal shape. Compression forces are positive and positive displacement corresponds to the retraction of the AFM probe from the substrate.

The critical point in any affinity atomic-force microscopy experiment is the assignment of any given F–z curve as resulting from either a genuine sensor molecule–target molecule interaction or from an unspecific event. In addition, this assignment should be self-contained and not require additional validation through a second experimental technique. In this regard, as discussed below, genuine LDH–anti-LDH interactions were established by considering three criteria: (i) the number of peaks, (ii) the value of the adhesion force (defined by the minimum of the F–z curve), and (iii) the presence or absence of the elastomeric trait in the region prior to the peak(s). Following these three criteria, the F–z curves recorded from the affinity microscopy tests using LDH-decorated MicroDeck^TM^ surfaces and anti-LDH antibody-decorated DeepTip^TM^ probes were classified into seven types, of which six are illustrated in Figure 4: Type 1 (no interaction), Type 2 (elastomeric, one peak), Type 3 (non-elastomeric, one peak), Type 4 (double peak, elastomeric), Type 5 (double peak, combined elastomeric/non-elastomeric), Type 6 (threefold or fourfold peaks), and Type 7 (discarded curves due to their abnormal shape; not shown in Figure 4).

**Figure 5:**
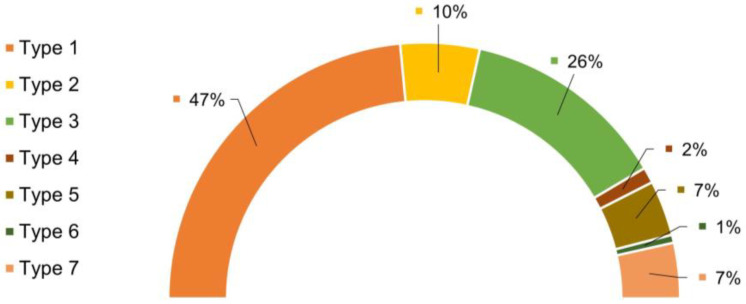
Percentages of each type of force–distance curve. (Type 1: 47%—no-interaction curves; Type 2: 10%—elastomeric curves; Type 3: 26%—non-elastomeric curves; Type 4: 2%—elastomeric–two-peak curves; Type 5: 7%—combined curves; Type 6: 1%—curves with threefold or fourfold elastomeric peaks; Type 7: 7%—discarded curves).

Based on this classification, the statistics that reflect the number of curves observed for each type of curve are shown in Figure 5. As seen in this figure, 47% corresponded to Type 1 (no interaction) and 7% corresponded to Type 7 (discarded curves). Correspondingly, a percentage slightly lower than 50% of the curves corresponded to events that might contain information on the interaction between a target molecule and a sensor molecule (antibody). Consequently, at this point, it is critical to identify those F–z curves that contain information on the genuine LDH–anti-LDH interactions. In this regard, it is worth highlighting the importance of the curves with multiple peaks and the elastomeric trait, corresponding to Types 4, 5, and 6. The elastomeric trait is identified in these curves by an increasing value of stiffness, i.e., an increasing value of the absolute value of the F–z slope in the region prior to the peak at increasing values of z. This fingerprint in the F–z curve is assumed to be related with the unfolding of the polyethyleneglycol (PEG) spacer through which the antibody is attached to the AFM probe, and corresponds to the characteristic mechanical behavior of polymer chains with a large conformational freedom (elastomeric behavior) [30].

In combination with the elastomeric trait, the variation in the value of the force after post-peak unloading is also a criterium for the proper identification of a genuine interaction, since it corresponds to the magnitude of the force lost when one or more bonds break. The curves in Figure 4 show that these values are close to 50 pN (Figure 4ii,iv–vi and Figure S1) and its multiples (e.g., 150 pN; Figure 4iv,vi and Figure S1). The presence in the same curve of two peaks with a maximum force of approx. 100 pN and it exhibiting the elastomeric trait expected from the unfolding of the PEG spacer is difficult to assign to two unrelated unspecific interaction events between the probe and the substrate. Following a previous work [19], this combination of features is taken as a reference to identify the genuine sensor–target interactions in a given F–z curve. Consistently with this discussion, the one-peak elastomeric curves are also considered as genuine sensor–target molecule interactions, taking advantage of the similarity of these peaks with those found in double- and multiple-peak curves, so that the percentage of F–z curves that reflect genuine interactions reaches at least 18% (Type 2 + Type 4 + Type 6) of the total number of curves.

Before starting the analysis of the non-elastomeric peaks, it is worth considering some general aspects of force–displacement curves in greater detail. To begin with, the importance of recording a sufficient number of no-interaction curves should be highlighted. In effect, no-interaction curves provide a self-consistency test to the whole experimental procedure. Thus, the observance of no-interaction curves distributed homogeneously during the recording of the F–z curves precludes the existence of a systematic bias of the data that might be related, for instance, to the contamination of the probe or to its interaction with the unquenched thiol sites on the substrate.

When antibodies are used as sensor molecules, the existence of curves with single, double, or multiple peaks exhibiting the elastomeric trait may be correlated to the structure of an antibody [31] in which two Fab regions are present that may bind the corresponding antigen independently. In this regard, single-peak curves can be interpreted as the binding of one Fab region of the anti-LDH antibody to the LDH protein. Following this interpretation, double, threefold, and fourfold peaks would correspond to the binding to the antigen of two, three, or four Fabs, respectively. This interpretation is coherent with the distribution of curves of each elastomeric type: one peak—Type 2 (10%) > two peaks—Type 4 (7%) > three and four peaks—Type 6 (1%), which reflects the decreasing probability of more than one Fab binding to the antigen. In this case, the interaction of both Fabs from two independent antibodies that may be bound to different sites of the same streptavidin molecule might account for a significant proportion of the four-peak/Type 6 curves.

At this point, it is worth mentioning that the proposed experimental procedure is designed to test the robustness of the whole scheme under favorable, but not optimal, conditions in terms of the interactions established between sensor and target molecules. Thus, no attempt was performed to control the orientation of the LDH while being immobilized, so there is no guarantee that some proteins would not present accessible epitopes that could be recognized by the antibody. In addition, as mentioned above, the conditions for the decoration of the substrates with LDH were selected to obtain a significant coverage of the surface, but were not intended for depositing a monolayer of protein on the material. In addition, it might possible to consider an alternative procedure based on the usage of individual Fabs instead of complete antibodies as sensor molecules. This latter approach may lead to a reduction in the variability of the adhesion peaks, but it might imply a decrease in the number of curves with double or multiple peaks, which are extremely helpful for distinguishing genuine from unspecific interactions. Lastly, it should be acknowledged that the usage of a polyclonal anti-LDH antibody presents certain limitations in terms of the reproducibility of the measured values of the adhesion forces, since the scattering of these values is likely to be larger than that found with the use of a monoclonal antibody. However, the possibility of obtaining reliable results in terms of the number of genuine sensor–target interactions recorded, in spite of using these non-fully optimized procedures, is a strong support to the robustness of the whole experimental approach.

Even if just those curves that present at least one elastomeric peak are considered, the ratio of successful interaction events reaches a value of 13%, significantly larger than the usual percentages of successful interaction events reported in other works, which are typically in the range of 0.1–1% [16,17]. This successful ratio is even higher (20%) if Type 5 curves (with one elastomeric/one non-elastomeric peak) are considered.

**Figure 6:**
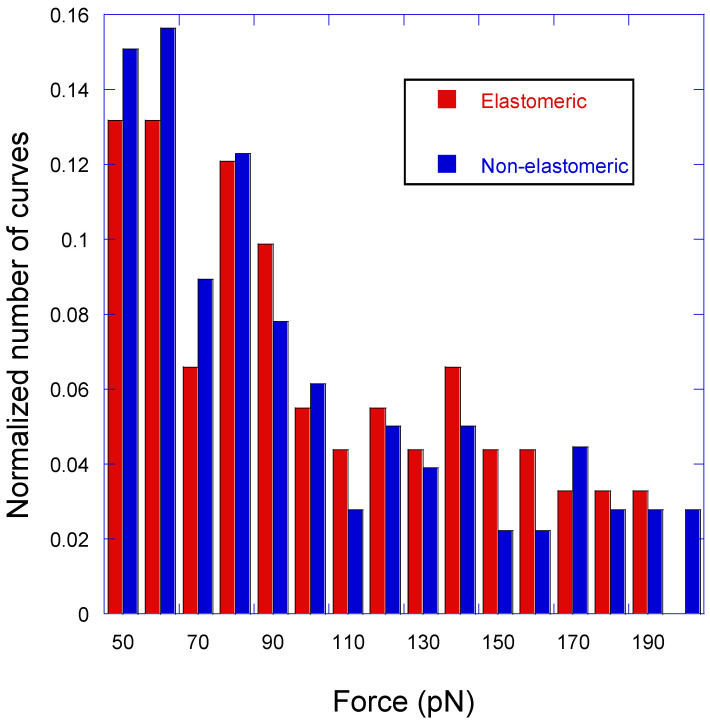
Statistical distribution of the force–distance curves corresponding to elastomeric and non-elastomeric peaks as a function of the measured adhesion force.

In order to interpret the significance of non-elastomeric curves, and following a previous work [19], Figure 6 shows the distribution of peaks with either the elastomeric or non-elastomeric trait in the range of forces between 50 pN and 200 pN. Although it may be argued that peaks with lower values of adhesion force may also correspond to specific interactions, while reflecting different pulling geometries [16], it cannot be completely discarded that these low-intensity peaks might result from other events different to a genuine LDH–anti-LDH interaction. This uncertainty led to the exclusion of the curves with low-intensity peaks from the ensuing statistical analysis. Alternatively, it might be also argued that peaks with higher adhesion forces do correspond to this specific interaction for single or multiple events. However, the justification of these high values of adhesion forces will require an additional analysis that is outside the scope of this work. In addition, high adhesion forces are scattered from the chosen upper limit at 200 pN up to values in excess of 1000 pN, which severely limits the reliable application of the goodness-of-fit test.

In this context, an interpretation of the non-elastomeric peaks (Type 2 and Type 5 curves) can be established by comparing the distribution of both types of peaks normalized to the total number of curves of each type. It can be assumed that the concurrence of both distributions supports the genuine character of the non-elastomeric peaks as resulting from the LDH–anti-LDH antibody interaction, since otherwise it would be implied that two unrelated events (specific target–sensor molecule interactions and other unspecific interactions) would yield a common statistical distribution. As described elsewhere, [19], the distribution of the non-elastomeric curves may be taken as a reference and compared with that of the elastomeric curves. The choice of the distribution of non-elastomeric curves as a reference distribution is suggested by the larger number of this type of curves (by over three times) with respect to the elastomeric ones. The comparison of both distributions follows the χ^2^ test of goodness of fit and starts by dividing the range of adhesion forces between 50 pN and 200 pN in 10 pN intervals. The value of the X^2^ parameter for the distribution of elastomeric curves using the distribution of non-elastomeric curves as a reference is X^2^ = 9.9, which is to be compared with the value χ^2^(f = 15) = 25.0 for the 15 degrees of freedom considered (the number of intervals in which the range of forces is divided minus one). Since χ^2^(f = 15) = 25.0 > X^2^ (=9.9), it is concluded that both sets of events correspond to the same statistical distribution. This concurrence of statistical distributions supports the assignment of non-elastomeric peaks to genuine LDH–anti-LDH antibody interactions, which implies that there is a percentage of almost 50% of successful events for the experimental procedure presented in this work.

## 4. Conclusions

In this study, a robust procedure for the decoration of DeepTip^TM^ AFM probes with biotinylated antibodies is presented and validated using LDH and an anti-LDH antibody as a model system. The crosslinking chemistries used for the immobilization of both molecules to the corresponding surfaces are presented in detail, and shown to be compatible with a wide range of biological and biomimetic systems. The interaction between the antibody (sensor molecule) and the LDH protein (target molecule) is analyzed by means of force–displacement (F–z) curves, and it is found that single-molecule interaction events can be efficiently recorded. F–z curves that correspond to a genuine sensor–target molecule interaction are identified from three basic criteria: the number of peaks, the magnitude of the adhesion forces, and the presence of the elastomeric trait. Following these criteria, the curves are classified into seven different types. A complete statistical analysis of the F–z curves yields that approximately 50% of the curves show events that reflect a genuine interaction between the sensor and target molecules. The high proportion of force–displacement curves that reflect genuine interactions between the sensor and target molecules, in combination with a versatile procedure that allows the covalent immobilization of antibodies to the AFM probe, increase considerably the number of systems that may be explored through affinity microscopy. In effect, this high yield of successful events decreases proportionally the number of force–displacement curves that need to be recorded to explore a given area of the sample in a typical affinity atomic-force microscopy experiment. The usage of biotinylated antibodies implies that the whole procedure can be implemented with minimum variations to a vast number of target molecules.

## Data Availability

Data can be retrieved upon request to the corresponding author.

## Supplementary Materials

The following supporting information can be downloaded at https://www.mdpi.com/article/10.3390/biomimetics9040192/s1: Figure S1: Topographic micrograph of a substrate after being incubated with an LDH solution. Figure S2: Example of an F–z curve with three peaks exhibiting the elastomeric trait in the region adjacent to the peak.

## References

[1] Binnig G., Quate C.F., Gerber C. (1986). Atomic Force Microscope. Anonymous Scanning Tunneling Microscopy.

[2] Eroles M., Lopez-Alonso J., Ortega A., Boudier T., Gharzeddine K., Lafont F., Franz C.M., Millet A., Valotteau C., Rico F. (2023). Coupled mechanical mapping and interference contrast microscopy reveal viscoelastic and adhesion hallmarks of monocyte differentiation into macrophages. Nanoscale.

[3] Martín-González N., Hernando-Pérez M., Condezo G.N., Pérez-Illana M., Šiber A., Reguera D., Ostapchuk P., Hearing P., Martín C.S., de Pablo P.J. (2019). Adenovirus major core protein condenses DNA in clusters and bundles, modulating genome release and capsid internal pressure. Nucleic Acids Res..

[4] Rigato A., Miyagi A., Scheuring S., Rico F. (2017). High-frequency microrheology reveals cytoskeleton dynamics in living cells. Nat. Phys..

[5] Zhang Q., Yang J., Tillieux S., Guo Z., Natividade R.D.S., Koehler M., Petitjean S., Cui Z., Alsteens D. (2022). Stepwise Enzymatic-Dependent Mechanism of Ebola Virus Binding to Cell Surface Receptors Monitored by AFM. Nano Let..

[6] Viljoen A., Mathelié-Guinlet M., Ray A., Strohmeyer N., Oh Y.J., Hinterdorfer P., Müller D.J., Alsteens D., Dufrêne Y.F. (2021). Force spectroscopy of single cells using atomic force microscopy. Nat. Rev. Methods Primers.

[7] Delguste M., Zeippen C., Machiels B., Mast J., Gillet L., Alsteens D. (2018). Multivalent binding of herpesvirus to living cells is tightly regulated during infection. Sci. Adv..

[8] Milles L.F., Schulten K., Gaub H.E., Bernardi R.C. (2018). Molecular mechanism of extreme mechanostability in a pathogen adhesin. Science.

[9] Chung J.W., Shin D., Kwak J.M., Seog J. (2013). Direct force measurement of single DNA-peptide interactions using atomic force microscopy. J. Mol. Recognit..

[10] Muddassir M., Manna B., Singh P., Singh S., Kumar R., Ghosh A., Sharma D. (2018). Single-molecule force-unfolding of titin I27 reveals a correlation between the size of the surrounding anions and its mechanical stability. Chem. Commun..

[11] Milles L., Gaub H. (2019). Is mechanical receptor ligand dissociation driven by unfolding or unbinding?. BioRxiv.

[12] Bergkvist M., Cady N.C. (2011). Chemical Functionalization and Bioconjugation Strategies for Atomic Force Microscope Cantilevers. Bioconjug. Protoc..

[13] Volcke C., Gandhiraman R.P., Gubala V., Doyle C., Fonder G., Thiry P.A., Cafolla A.A., James B., Williams D.E. (2010). Plasma functionalization of AFM tips for measurement of chemical interactions. J. Colloid Interface Sci..

[14] Barattin R., Voyer N. (2008). Chemical modifications of AFM tips for the study of molecular recognition events. Chem. Commun..

[15] Wildling L., Unterauer B., Zhu R., Rupprecht A., Haselgrübler T., Rankl C., Ebner A., Vater D., Pollheimer P., Pohl E.E. (2011). Linking of Sensor Molecules with Amino Groups to Amino-Functionalized AFM Tips. Bioconjug. Chem..

[16] Sedlak S.M., Schendel L.C., Gaub H.E., Bernardi R.C. (2020). Streptavidin/biotin: Tethering geometry defines unbinding mechanics. Sci. Adv..

[17] Guo S., Ray C., Kirkpatrick A., Lad N., Akhremitchev B.B. (2008). Effects of Multiple-Bond Ruptures on Kinetic Parameters Extracted from Force Spectroscopy Measurements: Revisiting Biotin-Streptavidin Interactions. Biophys. J..

[18] Corregidor D., Tabraue R., Colchero L., Daza R., Elices M., Guinea G.V., Pérez-Rigueiro J. (2023). High-Yield Characterization of Single Molecule Interactions with DeepTip[sup.TM] Atomic Force Microscopy Probes. Molecules.

[19] Daza R., Garrido-Arandia M., Corregidor-Ortiz D., Pérez C.I., Colchero L., Tabraue-Rubio R., Elices M., Guinea G.V., Diaz-Perales A., Pérez-Rigueiro J. (2023). Statistical Study of Low-Intensity Single-Molecule Recognition Events Using DeepTip^TM^ Probes: Application to the Pru p 3-Phytosphingosine System. Biomimetics.

[20] Hnasko R. (2015). Elisa.

[21] Kurien B.T., Scofield R.H. (2006). Western blotting. Methods.

[22] Urh M., Simpson D., Zhao K. (2009). Chapter 26 Affinity Chromatography: General Methods. Meth. Enzymol..

[23] Le T., Chang P., Benton D.J., McCauley J.W., Iqbal M., Cass A.E. (2017). Dual recognition element lateral flow assay (DRELFA) towards multiplex strain-specific influenza virus detection. Anal. Chem..

[24] Harada Y., Kuroda M., Ishida A. (2000). Specific and Quantized Antigen—Antibody Interaction Measured by Atomic Force Microscopy. Langmuir.

[25] Ouerghi O., Touhami A., Othmane A., Ouada H.B., Martelet C., Fretigny C., Jaffrezic-Renault N. (2002). Investigating antibody–antigen binding with atomic force microscopy. Sens. Actuators B Chem..

[26] Hinterdorfer P., Dufrêne Y.F. (2006). Detection and localization of single molecular recognition events using atomic force microscopy. Nat. Methods.

[27] Marcuello C., de Miguel R., Lostao A. (2022). Molecular Recognition of Proteins through Quantitative Force Maps at Single Molecule Level. Biomolecules.

[28] Arroyo-Hernández M., Daza R., Pérez-Rigueiro J., Elices M., Nieto-Márquez J., Guinea G.V. (2014). Optimization of functionalization conditions for protein analysis by AFM. Appl. Surf. Sci..

[29] Horcas I., Fernández R., Gómez-Rodríguez J.M., Colchero J., Gómez-Herrero J., Baro A.M. (2007). WSXM: A software for scanning probe microscopy and a tool for nanotechnology. Rev. Sci. Instrum..

[30] Pérez Rigueiro J. (2023). Biological Materials and Biomaterials.

[31] Abbas A.K., Lichtman A.H., Pillai S. (2018). Cellular and Molecular Immunology.

