# Supplementary material for: Identification of Individual Target Molecules Using Antibody-Decorated DeepTipTM Atomic-Force Microscopy Probes
Source: Biomimetics (Basel). 2024 Mar 22;9(4):192. doi: 10.3390/biomimetics9040192 (PMC11048431; doi:10.3390/biomimetics9040192)

biomolecules

Supplementary Figure S1

Representative topographic atomic force microscopy micrograph of a monocrystalline silicon substrate incubated for 1 h with 110 μg/mL of LDH solution in Tris-HCl (100 mM, pH 7.1). The root mean square deviation (RMS) of the image is 2.6 nm and may be compared with the RMS value of the monocrystalline, atomic-flat silicon substrate observed in the same buffer, RMS [Si]= 1.9 nm.

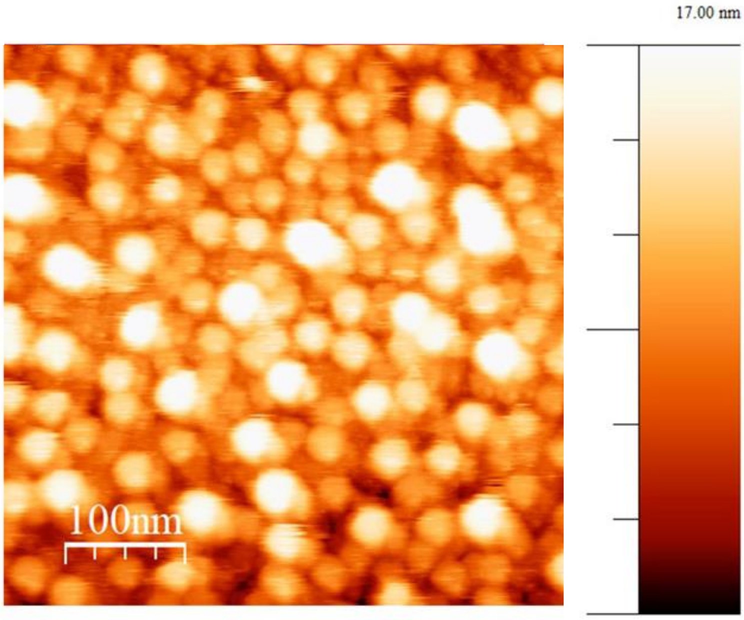

Supplementary Figure S2

Example of a F-z curves with three peaks exhibiting the elastomeric trait in the region adjacent to the peak.

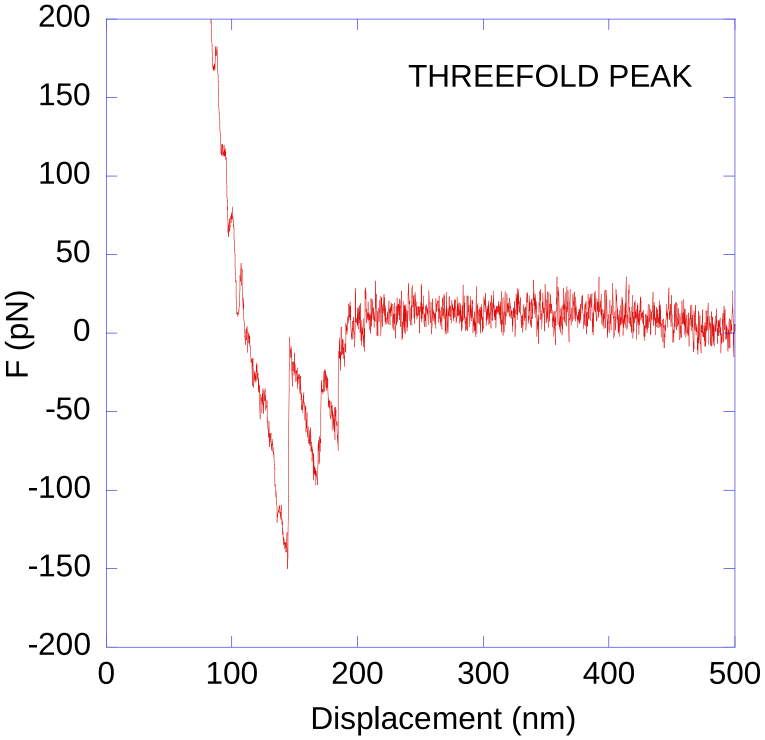

Supplement: Supplementary file 1 [file biomimetics-09-00192-s001.zip › biomimetics-2862927-supplementary.pdf]
